# Antioxidant Secondary Metabolites in Cereals: Potential Involvement in Resistance to *Fusarium* and Mycotoxin Accumulation

**DOI:** 10.3389/fmicb.2016.00566

**Published:** 2016-04-22

**Authors:** Vessela Atanasova-Penichon, Christian Barreau, Florence Richard-Forget

**Affiliations:** MycSA, Institut National de la Recherche AgronomiqueVillenave d'Ornon, France

**Keywords:** *Fusarium*, mycotoxins, cereals, antioxidants, resistance

## Abstract

*Gibberella* and *Fusarium* Ear Rot and *Fusarium* Head Blight are major diseases affecting European cereals. These diseases are mainly caused by fungi of the *Fusarium* genus, primarily *Fusarium graminearum* and *Fusarium verticillioides*. These *Fusarium* species pose a serious threat to food safety because of their ability to produce a wide range of mycotoxins, including type B trichothecenes and fumonisins. Many factors such as environmental, agronomic or genetic ones may contribute to high levels of accumulation of mycotoxins in the grain and there is an urgent need to implement efficient and sustainable management strategies to reduce mycotoxin contamination. Actually, fungicides are not fully efficient to control the mycotoxin risk. In addition, because of harmful effects on human health and environment, their use should be seriously restricted in the near future. To durably solve the problem of mycotoxin accumulation, the breeding of tolerant genotypes is one of the most promising strategies for cereals. A deeper understanding of the molecular mechanisms of plant resistance to both *Fusarium* and mycotoxin contamination will shed light on plant-pathogen interactions and provide relevant information for improving breeding programs. Resistance to *Fusarium* depends on the plant ability in preventing initial infection and containing the development of the toxigenic fungi while resistance to mycotoxin contamination is also related to the capacity of plant tissues in reducing mycotoxin accumulation. This capacity can result from two mechanisms: metabolic transformation of the toxin into less toxic compounds and inhibition of toxin biosynthesis. This last mechanism involves host metabolites able to interfere with mycotoxin biosynthesis. This review aims at gathering the latest scientific advances that support the contribution of grain antioxidant secondary metabolites to the mechanisms of plant resistance to *Fusarium* and mycotoxin accumulation.

## Introduction

*Fusarium* Head Blight (FHB) of small-grain cereals such as wheat and barley and *Gibberella* Ear Rot (GER) and *Fusarium* Ear Rot (FER) of maize are three devastating fungal diseases affecting crops worldwide. Both FHB and GER are caused by the same *Fusarium* species on wheat and maize respectively, *Fusarium graminearum* and *Fusarium culmorum* being the most predominant in Europe (Bottalico and Perrone, 2002). FER is caused by *Fusarium* species belonging to the *Gibberella fujikuroi* complex, including *Fusarium proliferatum* and *Fusarium verticillioides*. These three fungal diseases lead to huge economic losses, resulting from reduced yields, deteriorated grain quality and contamination of grains with mycotoxins.

*F. graminearum* and *F. culmorum* can produce zearalenone and type B trichothecenes (TCTB). TCTB include deoxynivalenol (DON) and its two acetylated forms, 3-acetyl-deoxynivalenol (3-ADON) and 15-acetyl-deoxynivalenol (15-ADON), as well as nivalenol (NIV) and its acetylated form 4-acetylnivalenol or fusarenon X (FX). *F. proliferatum* and *F. verticillioides* are major sources of maize contamination with fumonisins (FB) among which fumonisin B1 (FB1), FB2 and FB3 are predominant. All these *Fusarium* toxins exhibit various acute and chronic effects on humans and animals (Bennett and Klich, 2003). Consequently, thresholds for maximal DON, FB1+FB2 and zearalenone content in foodstuffs have been set up in Europe: Commission regulation published in 2005 (EC number 856/2005) and amended in July 2007 (EC number 1126/2007) for DON and zearalenone and Commission regulation number 1126/2007 for FB1+FB2. Published surveys on the mycotoxin status of European cereals and derived products clearly show that mycotoxins produced by *Fusarium* species are ubiquitously present and that contamination levels exceeding the EU maximum levels or guidance values are likely to occur, leading to significant economic losses (Streit et al., 2012; Schatzmayr and Streit, 2013; Nordkvist and Haggblom, 2014). There are no data on the economic costs of mycotoxins in Europe with the exception of one study in Hungary where these costs were estimated to be 100 million euros in 1998, consecutively to a severe FHB outbreak (Milicevic et al., 2010). Economic costs directly result from: (1) yield loss due to fungal diseases, (2) reduced crop value resulting from mycotoxin contamination, (3) losses in animal productivity and (4) trade impacts. Additional costs include the cost of management linked to prevention, sampling, analysis, mitigation, litigation, and research. For instance, the annual cost for monitoring aflatoxin alone in the US is estimated to be 30–50 million dollars.

Production of DON, FB and zearalenone by *Fusarium* spp. occurs during infection of crops. Structurally, DON is defined as an epoxide containing sesquiterpenoid skeleton. The epoxide group at position 12–13 allows DON to bind to ribosomes leading to the activation of various protein kinases, the modulation of gene expression, the inhibition of protein synthesis and cell toxicity (Maresca, 2013). The chemical structure of FB consists of an aminopentol backbone with one tricarballylic acid on each side chain and one or more hydroxyl groups (Bezuidenhout et al., 1988). Due to their structural similarity with sphinganine, FB may act as specific inhibitors of sphingolipid biosynthesis, which are major constituents of cell membranes and important components of many signaling pathways (Merrill et al., 2001). Zearalenone is a phenolic resorcyclic acid lactone and its toxicity is mainly related to its ability to competitively bind to estrogen receptors. Excellent reviews describing the detailed biosynthesis pathway for DON and FB have been recently published (Brown et al., 2007; Alexander et al., 2009) whilst there are still significant knowledge gaps in the understanding of zearalenone biosynthesis. During the last decades, tremendous progress has also been made in identifying the environmental factors that significantly impact the regulation of DON and FB biosynthesis during the colonization of plant tissues (Picot et al., 2010; Merhej et al., 2011; Montibus et al., 2015). Temperature, water availability, pH variations, nutrient sources and plant defense metabolites were pointed out as key factors regulating DON and FB production.

TCTB and FB toxins are heat-stable molecules that are not fully eliminated during food and feed processing (Hazel and Patel, 2004; Humpf and Voss, 2004). As a result, the best way to reduce or avoid contamination of food and feed is to control the biosynthesis of these mycotoxins at the field level during plant cultivation. Three major factors have been reported to significantly influence fungal development and mycotoxin accumulation in grains: (i) environmental conditions, (ii) agricultural practices, and (iii) susceptibility of cereal genotypes (Edwards, 2004). Several cultural practices such as crop rotation, tillage, use of chemicals as well as breaking the fungal disease cycle by adapting the sowing period or using resistant hosts have been shown to reduce efficiently the level of primary pathogen inoculum (Pirgozliev et al., 2003). More recently, integrated management studies have demonstrated the improvements that can be gained by combining multiple control strategies (Blandino et al., 2012). Plant breeding strategies are among the most promising and performing approaches to durably fight against *Fusarium* diseases and the contamination of cereals with mycotoxins. Undoubtedly, such strategies will be among the most important pillars of any integrated disease management programs (Terzi et al., 2014).

Plant resistance to *Fusarium* and mycotoxin accumulation is a highly complex mechanism. Five major types of resistance have been classified for wheat, and are transferable for barley and maize. However, mechanisms associated with one of these five types can be host specific. In wheat and barley, type I resistance operates against initial infection of the floret (Schroeder and Christensen, 1963), and in maize, it may be associated with silk resistance. Type II resistance limits spreading of the infection within the host. Unlike in wheat, fungal infection in barley usually does not spread from initially infected spikelets to adjacent spikelets. Type II resistance has therefore little meaning for barley. Type III concerns resistance to grain infection; type IV, tolerance and ability to maintain yields and finally type V resistance gathers all mechanisms of resistance to mycotoxin accumulation (Miller et al., 1985; Mesterhazy, 1995, 2002). Boutigny et al. (2008) proposed to divide the type V resistance into two components. The first one, called type V-1, represents resistance to toxin accumulation operated by metabolic transformation involving biochemical modification catalyzed by enzymes such as UDP-glycosyltransferases, gluthatione-S-transferases or cytochrome P450 mono-oxygenases (Karlovsky, 2011; De Boevre et al., 2014). The second one (type V-2) corresponds to resistance *via* inhibition of mycotoxin biosynthesis through the action of plant endogenous compounds. These compounds include both constitutively synthetized compounds and those induced in response to pathogen infection.

In addition to genetic approaches aiming at identifying and characterizing Quantitative Trait Loci (QTL) for FHB, FER and GER resistance, recent biochemical studies have been attempted to decipher the biochemical defenses that contribute to FHB, FER, and GER resistance and low mycotoxin accumulation. Mainly based on comparative approaches of metabolite composition of resistant and susceptible varieties, challenged or not with *Fusarium*, these attempts have implemented targeted analytical approaches and non-targeted global metabolomic developments (Siranidou et al., 2002; Bollina et al., 2011; Atanasova-Penichon et al., 2012; Picot et al., 2013; Sampietro et al., 2013; Gunnaiah and Kushalappa, 2014). A large set of metabolites potentially acting in cereals to counteract toxigenic *Fusaria* and reduce mycotoxin accumulation has been highlighted by these studies. These metabolites derive from primary and secondary plant metabolism and can be roughly classified in six major groups: fatty acids, amino acids and derivatives, carbohydrates, amines and polyamines, terpenoids and phenylpropanoids (Gauthier et al., 2015). Plant secondary metabolites with antioxidant properties, mainly terpenoids and phenylpropanoids are among the most frequently reported for their potential involvement in plant defense against fungal pathogens (Balmer et al., 2013). In addition to their key role as plant defense mediators and their participation to cell wall reinforcement, these compounds display antifungal properties and some of them can interfere with mycotoxin biosynthesis (Gauthier et al., 2015).

Here, we review the latest scientific advances that support the potential contribution of grain antioxidant secondary metabolites to cereal resistance to *Fusarium* and mycotoxin accumulation focusing on (i) *in vitro* studies on the effect of antioxidants on fungal development and mycotoxin production by *Fusarium*, (ii) identification of the major antioxidant metabolites that *Fusarium* can encounter during ear infection process, from anthesis to grain maturity, and (iii) relation between resistance to *Fusarium* and antioxidant content in cereals.

## Principal antioxidant secondary metabolites in cereals

In cereals, the main secondary metabolites with antioxidant activity belong to three groups including phenolic compounds, carotenoids and tocopherols (Boutigny et al., 2008). An additional group, consisting of benzoxazinoid derivatives, less abundant in grains but with multiple recognized biological activities, needs also to be addressed.

### Phenylpropanoids

Phenolics are considered the major contributors to total antioxidant capacity of cereal grains (Awika et al., 2003; Gorinstein et al., 2008). Phenolic compounds derive from the phenylpropanoid pathway and are divided into two groups: flavonoid phenylpropanoids including flavones, flavonols, flavanones, flavanols, anthocyanins and chalcones, and non-flavonoid phenylpropanoids such as stilbenes, lignans, and phenolic acids.

#### Non-flavonoid phenylpropanoids

Among non-flavonoid phenylpropanoids, phenolic acids are predominant in cereals (Dykes and Rooney, 2007; Gauthier et al., 2015). Phenolic acids are derivatives of either benzoic or cinnamic acids. In cereals, benzoic acid derivatives include gallic, *p*-hydroxybenzoic, vanillic, syringic, and protocatechuic acids while cinnamic acid derivatives include caffeic, chlorogenic, *p*-coumaric, sinapic, and ferulic acids. Phenolic acids found in cereals exist in both soluble (free) and insoluble (cell-wall-bound) forms. Soluble phenolic acids are either free acids or esterified to sugar conjugates. Insoluble phenolic acids are linked to various polysaccharides and to lignin through ester and ether bonds. Soluble forms are compartmentalized within the plant cell vacuoles and insoluble forms are distributed in cell walls. Phenolic compounds are concentrated in the outer layers of the grain, the pericarp and the aleurone, and in the germ, and are less abundant in the endosperm (Bily et al., 2003; Das and Singh, 2015).

Studies comparing composition of phenolic compounds in cereals reveal significant differences between cereal types, within varieties as well as within grain fractions (Adom and Liu, 2002; Ndolo and Beta, 2014; Pihlava et al., 2015). This variability associated to the large set of extraction protocols and analytical procedures that can be used when addressing the phenolic composition of grains explains the frequent discrepancies observed in published data. For instance, in the study of Adom and Liu (2002), maize grains were reported as the richest in total phenolic acids, followed by wheat, oat and rice while, in the report of Irakli et al. (2012), the highest levels in both free and bound phenolic acids were found in oat, followed by maize, wheat and rice. Nevertheless, in all published studies, the major portion of phenolics in grains exists as bound forms: 85% in maize, 75% in oat and wheat and 62% in rice (Adom and Liu, 2002; Boz, 2015; Das and Singh, 2015).

**Figure 1:**
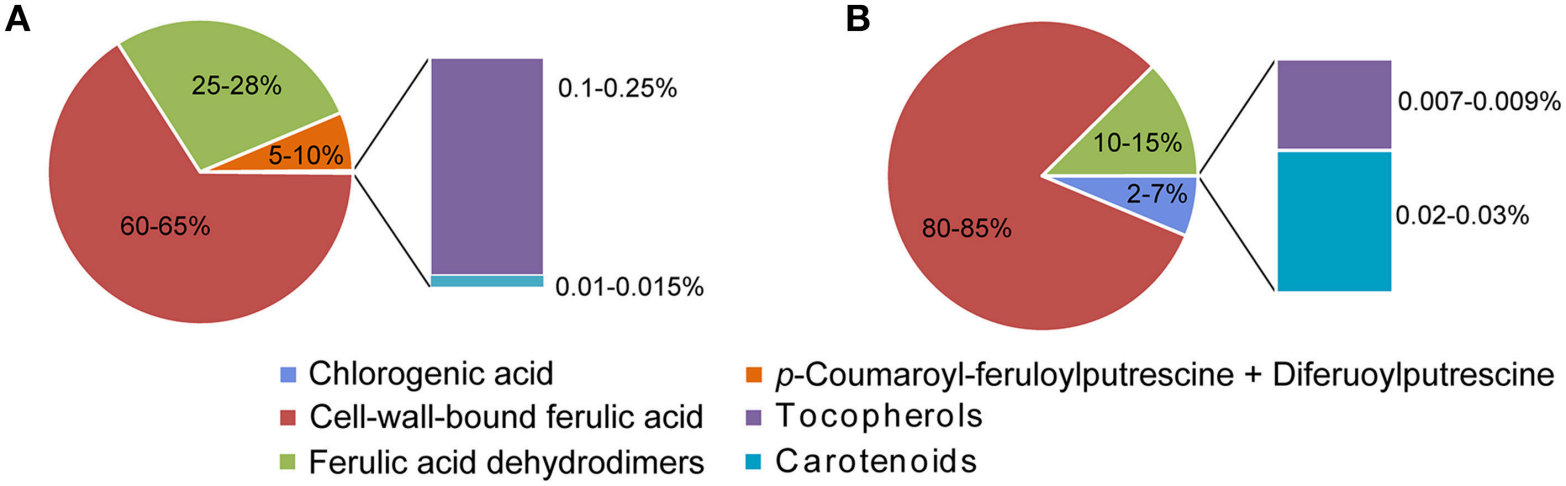
**Main antioxidant secondary metabolites quantified in maize at (A) maturity and at (B) early stage when mycotoxin biosynthesis is initiated (milk-dough stage)**. This model is based on maize results obtained by Atanasova-Penichon et al. (2012), Atanasova-Penichon and Richard-Forget (2014) and Picot et al. (2013).

Among free phenolic acids, ferulic acid is by far predominant, followed by *p*-coumaric and vanillic acids (Adom and Liu, 2002; Bakan et al., 2003; Santiago et al., 2007). Caffeic, *p*-hydroxybenzoic and sinapic acids are also present but at very low concentrations (0.5–1.5 μg/g) (Irakli et al., 2012). In addition to phenolic acid monomers, hydroxycinnamic polyamines such as *p*-coumaroyl-feruloylputrescine (CFP) and diferuoylputrescine (DFP) have been quantified in significant amounts in free phenolic maize extracts. Their concentrations can reach 330 μg equiv. 8-5′-benzofuran-diferulic acid/g (Moreau et al., 2001; Atanasova-Penichon et al., 2012; Figure 1A).

Among cell-wall-bound phenolic acids, ferulic acid is the most abundant one in common cereals and represents up to 90% of the total phenolic compounds (Adom and Liu, 2002; Boz, 2015). Concentrations of this compound can reach 3000 μg/g for some maize varieties (Li et al., 2007). Ferulic acid and its oxidatively coupled products named ferulic acid dehydrodimers or diferulic acids (DiFA) are found in greater concentrations in cereal brans. Ferulic acid dehydrodimers are potent antioxidants and are ester-linked to the cell wall polymers. The most important ferulic acid dehydrodimers in common cereals are: 8–5 DiFA (open form), 5-5′DiFA, 8-O-4′DiFA and 8-5′benDiFA (benzofuran form) and their sum represents 20–30% of the total phenolic acids (Atanasova-Penichon et al., 2012; Boz, 2015) (Figure 1A). The highest levels of ferulic acid dehydrodimers in cereals ranged between 250 and 475 μg/g (Jilek and Bunzel, 2013).

#### Flavonoid phenylpropanoids

The second group of phenolic compounds with significant concentrations in cereal grains is the class of flavonoids, located in the pericarp and the germ (Dykes and Rooney, 2007; Das and Singh, 2015). Flavonoids are also major active ingredient in corn silks (Hu et al., 2010). As phenolic acids, most grain flavonoids are found in the cell-wall-bound fraction: 93% of the total flavonoids in wheat, 91% in maize, 65% in rice and 61% in oat (Adom and Liu, 2002). According to Adom and Liu (2002), maize grains contain the highest level in total flavonoids followed by wheat, oat and rice. The most frequently cited flavonoids in cereal grains are the flavonols kaempferol and quercetin for maize (Das and Singh, 2015), the flavanone naringenin and its glycosylated forms and the flavanols catechin and epicatechin for barley (Bollina et al., 2010, 2011; Zilic et al., 2011), the flavones vitexin and luteolin for rye (Pihlava et al., 2015) and the anthocyanins in colored grains (Dykes and Rooney, 2007). According to the report of Reid et al. (1992), corn silks are characterized by high concentrations of flavones including luteolin and apigenin and flavone glycosides such as maysin, iso-orientin, and iso-vitexin.

### Lipophilic compounds

In cereals, the major lipophilic secondary metabolites with antioxidant properties include tocols (or commonly referred to as tocopherols) and carotenoids. The latest group consists of carotenes, of which α-carotene and β-carotene are the major representatives, and xanthophylls, mostly lutein and zeaxanthin. According to the results of two-year field studies, lipophilic antioxidant secondary metabolites represent less than 0.25% of total antioxidant secondary metabolites in mature maize grains (Atanasova-Penichon et al., 2012; Picot et al., 2013; Figure 1A). Concentrations in carotenoids in grains significantly vary according to the cereal type, from 1.8 μg/g in oat to 18.2 μg/g in maize (Ndolo and Beta, 2013). The major carotenoids in maize are concentrated in the endosperm fraction, ranging from 14.2 to 31.2 μg/g of endosperm, while the major carotenoids in small-grain cereals are found in germ and range from 3.2 to 14.8 μg/g of germ (Ndolo and Beta, 2013). In wheat, barley and oat grains, lutein was reported as the major xanthophyll and zeaxanthin as the minor one (Ndolo and Beta, 2013).

Tocol composition of cereals includes tocopherols (α-, β-, δ- and γ-tocopherol) and tocotrienols (α-, β-, δ- and γ-tocotrienols). The α-forms are predominant (Gutierrez-Gonzalez et al., 2013). Tocopherols are mainly present in the germ fraction while tocotrienols are present in the pericarp and endosperm fractions (Falk et al., 2004). In small-grain cereals such as oat, barley and wheat, tocotrienols are the main tocols and their concentrations range between 40 and 60 μg/g depending on the cereal type and the variety (Falk et al., 2004). Conversely, maize grains contain more tocopherols than tocotrienols, with concentrations ranging between 34–70 μg/g and 20–25 μg/g respectively (Das and Singh, 2015).

### Benzoxazinoid derivatives

Benzoxazinoids are a group of secondary metabolites found in maize, rye, wheat and triticale, but not in sorghum and rice (Niemeyer, 2009; Andersson et al., 2014). The mono and dihexose conjugates of cyclic hydroxamic acid DIBOA (2,4-dihydroxy-1,4-benzoxazin-3-one) and the corresponding lactam HBOA (2-hydroxy-1,4-benzoxazin-3-one) are the major benzoxazinoids in rye, while in wheat and maize, DIMBOA (2,4-dihydroxy-7-methoxy-1,4-benzoxazin-3-one) and its glycosylated derivative dominate (Etzerodt et al., 2015; Pihlava et al., 2015). Rye is by far the crop characterized by the highest content in benzoxazinoids, with concentration values more than 20-fold higher to that found in whole grain wheat (Andersson et al., 2014; Pihlava et al., 2015). Benzoxazinoids are located in all fractions of seeds, with greater concentrations in bran and germ (Pihlava et al., 2015).

## Antioxidants modulate fungal development and mycotoxin production by *Fusarium* spp.

The biosynthetic pathways that lead to the production of TCTB and FB by *Fusarium* species have been well established and characterized by several oxygenation steps (Proctor et al., 2003; Desjardins, 2007). Therefore, changes in the oxidative parameters of the medium are likely to interfere with the fungal secondary metabolism and to modulate the level of mycotoxin production (Ponts, 2005; Montibus et al., 2015). Due to the antioxidant properties of cereal secondary metabolites, several studies have been devoted to their anti-fungal and anti-mycotoxin effect.

### Antifungal properties of cereal antioxidants

Phenolic acids are toxic toward many fungi including *Fusarium* species (Guiraud et al., 1995; Ponts et al., 2011; Gauthier et al., 2016). Their fungicidal efficiency has been characterized against different *Fusarium* species and IC_50_ values (concentration that inhibits 50% of fungal growth) ranging between 0.7 to >10 mM have been reported (Table 1). The comparison of IC_50_ values has to be considered with caution as these values are method and condition dependent. IC_50_ values gathered in Table 1 also illustrate the great variability in phenolic acid sensitivity between *F. graminearum* strains. Similar variations seem to occur among *F. culmorum* strains; however there is insufficient data to assert this hypothesis. When comparing results obtained in the same conditions by Gauthier et al. (2016) and Ponts et al. (2011) for *F. graminearum* and *F. culmorum*, it also appears that *F. culmorum* strains (IC_50_ between 8.8 to >10.0 mM) could be less susceptible to caffeic acid than *F. graminearum* strains (IC_50_ between 4 to 10.1 mM). Phenolic acids could be ranked in ascending order of toxicity toward *F. graminearum* as follows: chlorogenic acid < *p*-hydroxybenzoic acid < caffeic acid < syringic acid < *p*-coumaric acid < ferulic acid (Table 1). Chlorogenic acid, a cinnamic-derived phenolic acid, displays a lower fungicidal activity than *p*-hydroxybenzoic and syringic acid, which is contradictory with the assumption that cinnamic-derived phenolic acids are roughly more toxic than benzoic acid-derived ones (Beekrum et al., 2003; Ponts et al., 2011). Chlorogenic acid displays however weak lipophilic properties, which, according to Guiraud et al. (1995) and Ponts et al. (2011) are primary factors in the antifungal efficiency of phenolic acids.

**Table 1:** **IC${}_{50}^{\text{a}}$ values of non-flavonoids and flavonoids against different *Fusarium* species**.

**Compound**	***Fusarium* species**	**IC_50_, mM**	**References**
**NON FLAVONOIDS: CINNAMIC ACID DERIVATIVES**			
Ferulic acid	*F. graminearum*	0.7–2.2	Ponts et al., 2011
	*F. graminearum*	1.8	Bollina et al., 2010
	*F. graminearum*	2.4	Kumaraswamy et al., 2011a
	*F. graminearum*	2.3, 3.4	McKeehen et al., 1999
	*F. culmorum*	1.7	McKeehen et al., 1999
	*F. coeruleum, F. moniliforme, F. solani*	5.2–>5.2	Guiraud et al., 1995
*p*-Coumaric acid	*F. graminearum*	1–4.2	Ponts et al., 2011
	*F. graminearum*	1.2	Bollina et al., 2010
	*F. graminearum*	1.9, 4.8	McKeehen et al., 1999
	*F. culmorum*	3.4	McKeehen et al., 1999
Caffeic acid	*F. graminearum*	4.0–7.1	Ponts et al., 2011
	*F. graminearum*	2.5	Kumaraswamy et al., 2011a
	*F. graminearum*	6.7–10.1	Gauthier et al., 2016
	*F. culmorum*	8.8–>10.0	Gauthier et al., 2016
Chlorogenic acid	*F. graminearum*	>10.0	Gauthier et al., 2016
	*F. culmorum*	>10.0	Gauthier et al., 2016
**NON FLAVONOIDS: BENZOIC ACID DERIVATIVES**			
*p*-Hydroxybenzoic acid	*F. graminearum*	6.6–>15.0	Ponts et al., 2011
Syringic acid	*F. graminearum*	3.5–6.2	Ponts et al., 2011
	*F. coeruleum, F. moniliforme, F. solani*	>5.0	Guiraud et al., 1995
Protocatechuic acid	*F. coeruleum, F. moniliforme, F. solani*	>6.5	Guiraud et al., 1995
Vanillic acid	*F. coeruleum, F. moniliforme, F. solani*	>6.0	Guiraud et al., 1995
**FLAVONOIDS: FLAVONES**			
Quercetin	*F. graminearum*	2.9	Bollina and Kushalappa, 2011
Kaempferol	*F. graminearum*	4.8	Bollina et al., 2010
**FLAVONOIDS: FLAVANONES**			
Flavanone	*F. graminearum, F. culmorum, F. poae*	<0.2	Silva et al., 1998
	*F. avenaceum, F. nivale*	<0.8	Silva et al., 1998
Naringenin	*F. graminearum*	1.6	Bollina et al., 2010
**FLAVONOIDS: FLAVONES**			
Flavone	*F. graminearum, F. culmorum, F. poae*	<0.2	Silva et al., 1998
	*F. avenaceum*	<0.8	Silva et al., 1998
	*F. nivale*	<0.05	Silva et al., 1998
4′-methylflavone	*F. graminearum, F. nivale, F. poae*	<0.2	Silva et al., 1998
	*F. culmorum, F. avenaceum*	>0.8	Silva et al., 1998
4′-methyoxyflavone	*F. graminearum, F. culmorum, F. avenaceum, F. nivale, F. poae*	>0.8	Silva et al., 1998

^a^Concentration that inhibits 50% of growth.

Compared to IC_50_ values ascribed to phenolic acids, those determined for flavones and flavanones against different *Fusarium* species including *F. culmorum* and *F. graminearum* are substantially weaker (Table 1). According to Treutter (2005), the efficiency to inhibit fungal growth directly results from the ability of flavonoids to irreversibly combine with nucleophilic amino acids in fungal proteins. Among the different groups of the flavonoid subclass, i.e., flavanones, flavones and flavanols, data reported in Table 1 suggest that unsubstituted flavones and flavanones (IC_50_ between < 0.05 to 1.6 mM) display a more efficient antifungal activity than hydroxylated flavones, i.e., flavonol (IC_50_ between 2.9 to 4.8 mM). The promising ability of flavonoids to inhibit spore development and restrain mycelium hyphae elongation of plant pathogens have been the subject of numerous investigations (Treutter, 2006; Mierziak et al., 2014). Flavonoids are also the subject of intensive medical research, with the aim of identifying alternatives to synthetic drugs for counteracting human fungal pathogens that increasingly display resistance to commonly used antifungal agents such as triazole ones (Cushnie and Lamb, 2005).

As regards to benzoxazinoids, their antifungal activities have been the subject of numerous publications (Glenn et al., 2001; Martyniuk et al., 2006). According to the results of Glenn et al. (2001), *Fusarium* species responsible for GER and FER in maize show a wide range of sensitivity to 6-methoxybenzoxazolin-2(3H)-one (MBOA) and benzoxazolin-2(3H)-one (BOA), with the most tolerant being *F. verticillioides, Fusarium subglutinans* and *F. graminearum*. As demonstrated by Glenn et al. (2001), differences in tolerance can be ascribed do different abilities to metabolize and therefore detoxify these antimicrobial compounds.

### Cereal antioxidants inhibit mycotoxin biosynthesis by *Fusarium*

In addition to displaying antifungal properties, several antioxidant secondary metabolites of cereals can modulate the production of mycotoxins by various fungal pathogens. According to the report of Boutigny (2007), cinnamic acid derivatives such as sinapic, caffeic, *p*-coumaric, chlorogenic, and ferulic acids are efficient inhibitors of TCTB production by *F. graminearum* and *F. culmorum* while benzoic acid derivatives, with the exception of syringic acid, have an activating effect. It is noteworthy that the effect of phenolic compounds is strain and molecule dependent (Boutigny et al., 2009; Gauthier et al., 2016). Increasingly, phenolic acids are becoming the subject of anti-mycotoxin research and many groups have demonstrated their efficiency to modulate *in vitro* the biosynthesis of various mycotoxins, including type A trichothecenes (Ferruz et al., 2016), fumonisins (Beekrum et al., 2003; Samapundo et al., 2007; Atanasova-Penichon et al., 2014), ochratoxin (Palumbo et al., 2007), and aflatoxins (Norton, 1999).

Similarly, several studies illustrated the potential impact flavonoids could exert on mycotoxin production. Recently, rutin was demonstrated as a potent inhibitor of aflatoxin B1 production by *Aspergillus flavus* (Norton, 1999; Chitarrini et al., 2014) and naringin, hesperidin and some glucosides were characterized for their capacity to restrain the production of patulin by *Penicillium expansum, Aspergillus terreus*, and *Byssochlamys fulva* (Salas et al., 2012). As regards to TCTB, effects of flavonoids on their biosynthesis have been poorly documented with exception of the publication of Desjardins et al. (1988) describing an inhibitory effect of flavones on the biosynthetic step that catalyzes the conversion of trichodiene (the first chemical intermediate in trichothecene biosynthesis) to oxygenated trichothecenes. In addition to phenolic compounds, carotenoids and tocopherols are potent cereal antioxidant compounds, but their antifungal and antimycotoxin activities against *Fusarium* are poorly documented. Recent works have shown that sub-lethal doses of α-tocopherol significantly affected fumonisin production (Picot et al., 2013) and that 50 μg/ml of β-carotene added to the culture medium led to a significant decrease (close to 50%) in TCTB accumulation (Boutigny, 2007). A few additional studies investigated the impact of carotenoids on other mycotoxin production but they led to opposite results, depending on the mycotoxin targeted. While capsanthin (a major carotenoid in paprika) has been shown to inhibit aflatoxin yield (Masood et al., 1994), more recent results demonstrated its lack of inhibitory effect on ochratoxin production (Santos et al., 2010).

The toxin suppressive effects of benzoxazinoids have also been addressed in several publications. This effect was first suggested by Miller et al. (1996) who reported that 4-acetyl-benzoxazolin-2-one (4-ABOA) and related compounds present in an active maize fraction were able to reduce trichothecene and aflatoxin productions by *F. culmorum* and *A. flavus*, respectively. Anti-mycotoxin activities of benzoxazinoids were recently confirmed by Etzerodt et al. (2015) who demonstrated that a 250 μM concentration of DIMBOA caused 50% inhibition of 15-ADON production by *F. graminearum*.

### Mechanisms of fungal toxicity and inhibition of mycotoxin production

Regardless the phytochemical considered (phenolic compound, carotenoid, tocopherol, and benzoxazinoid), the exact mechanisms by which fungal growth and mycotoxin production are inhibited remain unclear. While few published studies have focused on plant fungal pathogens, the mechanism of action of phenolic acids on human pathogens and mainly on *candida* species have been the subject of extensive research. Last and most significant insights on the anti-adhesion, anti-biofilm effects of phenolic acids together with their inhibitory activity on morphogenesis and fungal exoenzymes production have been recently gathered in the review of Teodoro et al. (2015). Interestingly, phenolic acids have been evidenced for their ability to breakdown the fungal membrane permeability barrier, probably through a perturbation of the lipid bilayers causing the leakage of ions and other chemicals as well as the formation of pores and modification of the electric potential of membranes (Sung and Lee, 2010).

As regards to the effects of phenolic acids against plant fungal pathogens, Guiraud et al. (1995) and more recently, Boutigny (2007) and Ponts et al. (2011) indicated that the toxicity of phenolic compounds is related to their lipophilicity as well as their strong antioxidant properties. Accordingly, Pani et al. (2014) and Roleira et al. (2010) suggested that the balance among lipophilicity and antioxidant activity can be a key factor to predict the capacity of a phenolic to inhibit mycotoxin production. However, it is essential to keep in mind that fungal cultures are multi-component systems, where the media can be considered as both lipidic and emulsion systems and that in such biological media, several physicochemical parameters including pH, light or temperature can affect the lipophilicity and antioxidant capacity of phytochemicals. Thus, correlating theoretical antioxidant potential and lipophilicity values with experimental data is not a straightforward approach. Nonetheless, the hypothesis that antioxidant properties of cereal metabolites can be primary factors for their antimycotoxin activity is highly consistent with the assumed activating effect of oxidative stress on the biosynthesis of mycotoxins. Indeed, an increasing body of work, recently gathered in the review of Montibus et al. (2015), emphasizes the modulation of fungal secondary metabolism by oxidative stress and the enhancement of mycotoxin production, including DON and FB, after exposure to reactive oxygen species. Thus, due to their capacity to quench oxygen free radicals, antioxidant metabolites may reduce or suppress upstream signals, such as oxidative stress, that modulate toxin biosynthesis. According to Guiraud et al. (1995), toxicity of phenolic acids can also be linked to their interaction with various intra or extracellular fungal enzymes, including phenol oxidases and several hydrolytic activities (El Modafar et al., 2000; Paul et al., 2003). Moreover, Passone et al. (2009) mentioned that antioxidant compounds interfere with mycotoxin production probably indirectly *via* their capacity to perturb the membrane function and modify its permeability. Lastly, the results of Boutigny et al. (2009) and Etzerodt et al. (2015) that indicate a downregulation of the expression of the genes involved in DON biosynthesis by *F. graminearum* when ferulic acid and DIMBOA is added to *in vitro* culture media are in accordance with a transcriptional control exerted by phenolic acids and benzoxazinoids. A similar conclusion was evidenced by the study of Sanzani et al. (2009) that proved that quercetin and umbelliferon reduced patulin accumulation by acting on the transcription level of biosynthetic genes.

## Antioxidant secondary metabolites encountered by *Fusarium* spp. during the ear infection process

**Figure 2:**
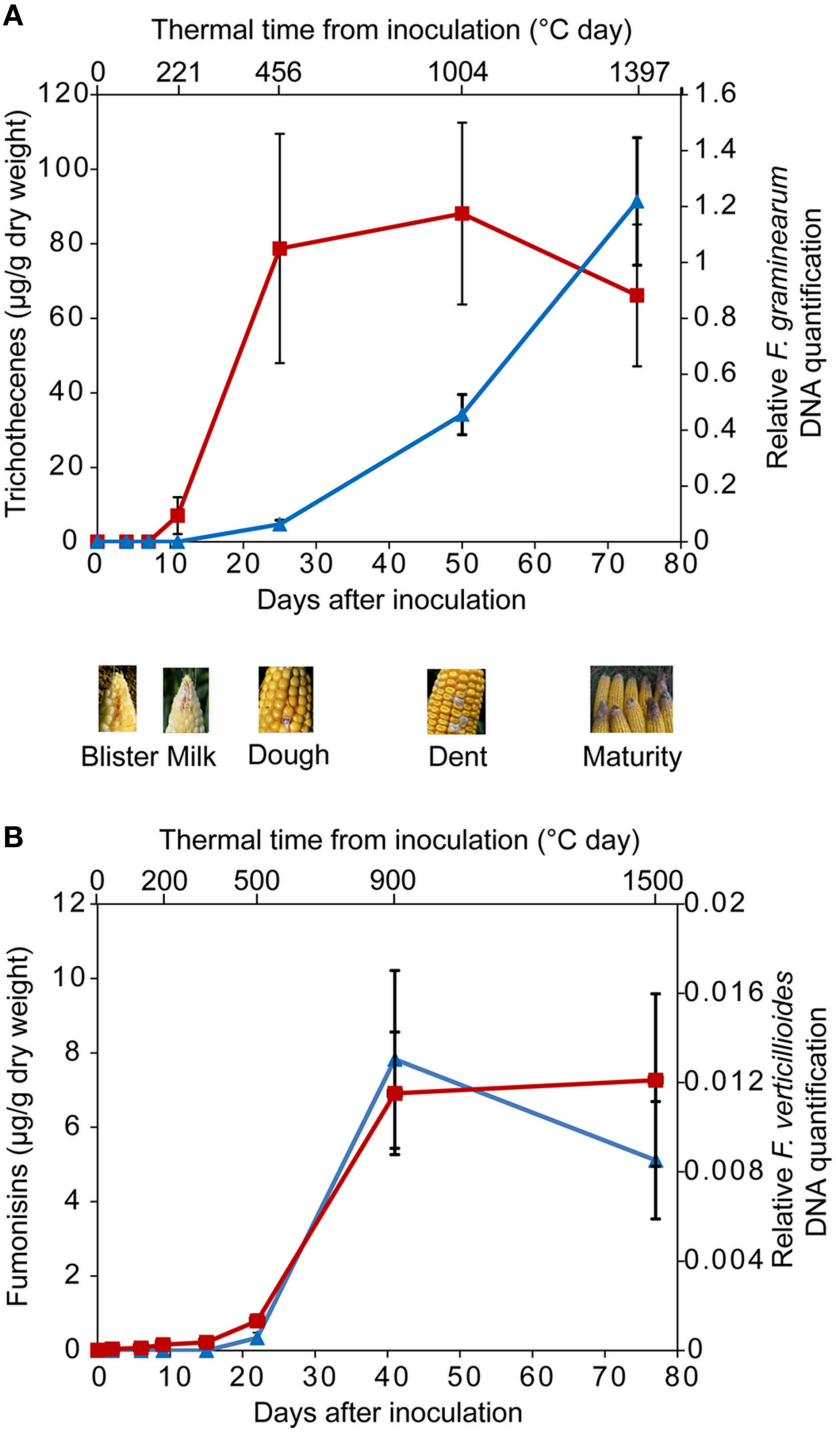
**(A)** Relative quantification of fungal DNA in maize kernels, expressed as a log10 (*F. graminearum* DNA/maize DNA) ratio of a susceptible variety (blue triangles, right Y-axis), and level of trichothecenes accumulated in maize kernels (red squares, left Y-axis) after silk inoculation with *F. graminearum*. Vertical bars show standard error of the mean. Top X-axis: thermal time from inoculation (mean value of 2 years), bottom X-axis: days after inoculation for each sampling (mean value of 2 years). Data from the 2 years and two repetitions were pooled (mean values ± SEM, *n* = 4). **(B)** Relative quantification of fungal DNA in maize kernels, expressed as a log10 *F. verticillioides* DNA/log10 maize DNA ratio of a susceptible variety (blue triangles, right Y-axis), and level of fumonisins accumulated in maize kernels (red squares, left Y-axis) after silk inoculation with *F. verticillioides*. Vertical bars show standard deviations. Top X-axis: thermal time from inoculation, bottom X-axis: days after inoculation for each sampling. Data from the two sowing date treatments were pooled. Kinetics are established with data published by Atanasova-Penichon et al. (2012) and Picot et al. (2011).

**Figure 3:**
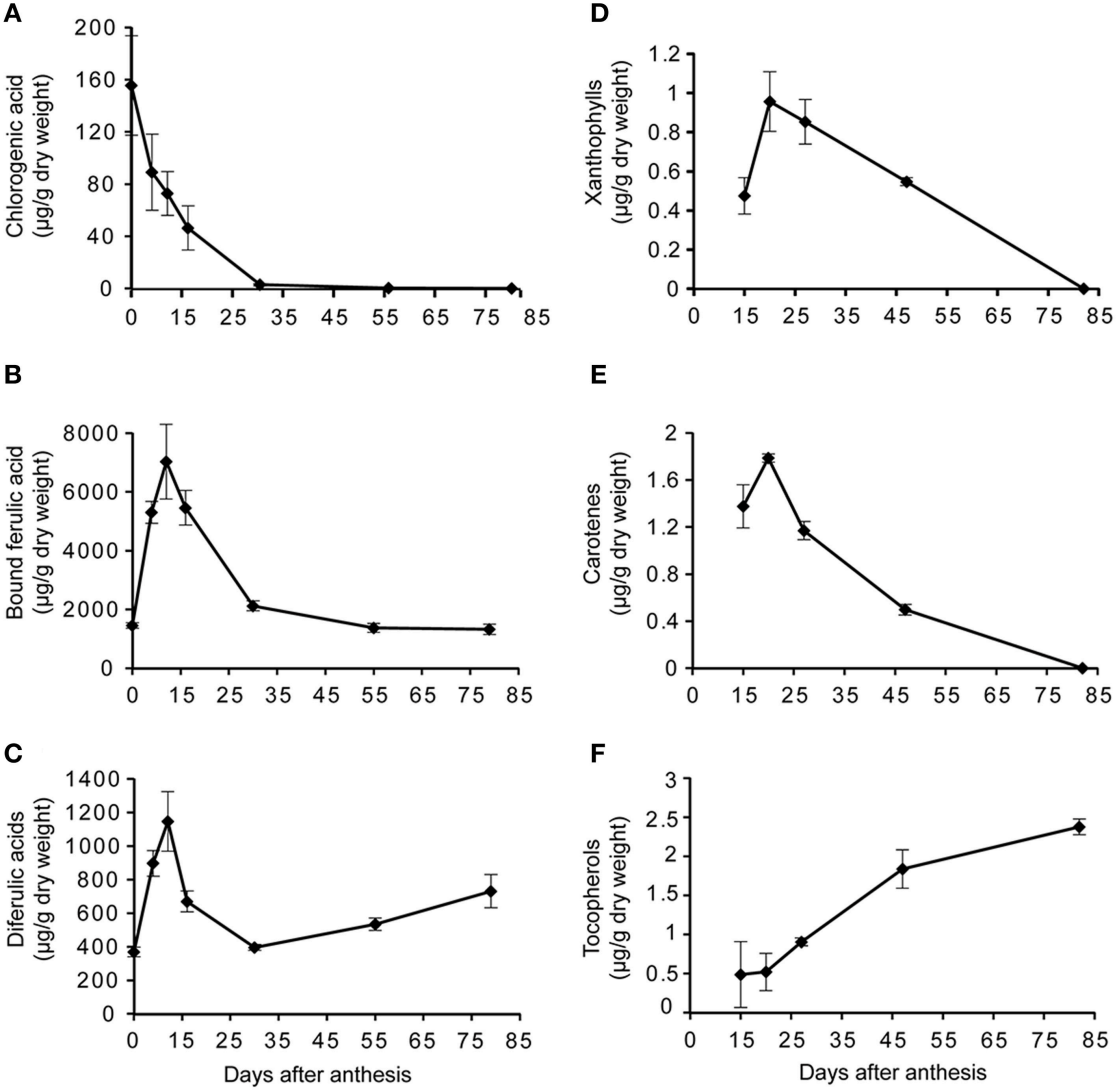
**Kinetics of antioxidant compounds during maize grain development: (A) chlorogenic acid; (B) cell-wall-bound ferulic acid; (C) ferulic acid dehydrodimers; (D) xanthophylls; (E) carotenes and (F) tocopherols**. Kinetics are established with data published by Atanasova-Penichon et al. (2012), Atanasova-Penichon and Richard-Forget (2014) and Picot et al. (2013).

To date, most of the attempts aiming at clarifying the contribution of cereal secondary metabolites to the *in planta* control of *Fusarium* mycotoxin accumulation have targeted mature grains. However, during plant development, grain antioxidant composition is likely to be dramatically modified. *Fusarium* commonly infects cereal ears shortly after anthesis, and the compounds the fungus has to face at the onset of infection are certainly extremely different from those found in the mature grain. There are very few dynamical studies that have addressed the composition of the grain in the early stages of grain development, when the biosynthesis of mycotoxin is initiated. In recent field experiments on maize inoculated with *F. graminearum* or *F. verticillioides*, the kinetics of fungal development and the accurate stage at which mycotoxin production is initiated were established (Picot et al., 2011, 2013; Atanasova-Penichon et al., 2012). *In planta* TCTB and FB accumulation were found to start between 10 and 20 days after flowering, i.e., at the milk-dough stage (Figure 2). Major free and bound antioxidant secondary metabolites present at the milk-dough stage were quantified and are detailed in Figure 1B. Cell-wall-bound ferulic acid, which represents 80–85% of the analyzed antioxidants, is the predominant compound, followed by ferulic acid dehydrodimers (10–15%) and free chlorogenic acid (2–7%). A particular attention was paid to free antioxidants, particularly to chlorogenic acid that represents almost 80% of the total free phenolic acids in the early stages of maize grain development. Indeed, free antioxidant compounds are more likely to interfere first with *Fusarium.* Phenolic compounds present in kernels at early stages are likely to alleviate fungal infection in a manner similar to that observed in the *in vitro* inhibition studies. In maize grains at the milk-dough stage, lipophilic antioxidants such as carotenoids and tocopherols are present at much lower levels than phenolic acids and represent only 0.02–0.03% of the total antioxidant content (Figure 1B). However, because their antioxidant properties are much higher than that of phenolic compounds, it cannot be excluded that, despite their low concentrations, they also significantly contribute *in planta* to the inhibition of *Fusarium* toxin biosynthesis. In addition to highlight the milk-dough stage as a critical step, the studies of Atanasova-Penichon et al. (2012) and Picot et al. (2013) provided information on the evolution of antioxidant secondary metabolites during maize ear ripening (Figure 3). Similar evolution patterns of phytochemicals were reported by the previous authors for the two years of experimentation, suggesting that they may correspond to an intrinsic characteristic of maize genotypes not dependent on environmental factors. Free and cell-wall-bound phenolic acid as well as carotenoid and tocopherol contents show large fluctuations during the ripening of maize grains. Composition in free phenolic acid evolves qualitatively over time whereas the composition in cell-wall-bound phenolic acids, carotenoids and tocopherols remains unchanged and only shows quantitative variation at the different grain stages. Kinetic of free chlorogenic acid, cell-wall-bound ferulic acid and ferulic acid dehydrodimers as well as xanthophylls, carotenes and tocopherols during maize ear ripening is presented in Figure 3. Except for tocopherols, all antioxidant secondary metabolites are found at higher concentrations in the grain at early stages, suggesting that these compounds are the main antioxidants that *F. graminearum* and *F. verticillioides* potentially encounter when their mycotoxin production is initiated. Figure 3 indicates that, after a rapid increase from anthesis to the silking-blister stage (with exception of chlorogenic acid), levels of cell-wall-bound monomers represented by ferulic acid, of free phenolic acids represented by chlorogenic acid, of xanthophylls and of carotenes decrease to reach traces at maturity. As regards the ferulic acid dehydrodimers, their concentration exhibits a pattern similar to monomeric phenolic acids in the first stages of grain development and then increases until the mature stage. This increase reflects the contribution of ferulic acid dehydrodimers to cell wall structure through their role in forming bridges between hemicellulose chains. A similar pattern for evolution of bound ferulic acid has also been reported for wheat (Shewry et al., 2012) and rice (Lin and Lai, 2011). Similarly, a decrease in free ferulic acid in rice (Lin and Lai, 2011) and total free phenols in oat (Alfieri and Redaelli, 2015) has been described, supporting old data on soft and durum wheat grain (McCallum and Walker, 1990; Régnier and Macheix, 1996; McKeehen et al., 1999). However, considering that most of the studies mentioned above were conducted with few genotypes, caution should be taken in generalizing the results.

The declining concentrations of phenolic acids during grain ripening can be ascribed to several rationales. First, the activity of phenyl-alanine ammonia-lyase and L-tyrosine ammonia-lyase, two crucial enzymes for the initial committed step in the biosynthesis of phenylpropanoids, have been shown to be maximal only during the early stages of grain development (McCallum and Walker, 1990; Régnier and Macheix, 1996). Second, the rate of endosperm development surpasses the rate of synthesis of the outer coverings during grain ripening which leads to a dilution of the overall phenolic constituents within the grain. Third, the decrease in phenolic acids can also result from their oxidative degradation involving phenoloxidases and peroxidases, induced by the breakdown of cellular structure in the pericarp at the end of the milk stage and during further maturation (Régnier and Macheix, 1996). Finally, the decrease in cell wall-bound phenolic acid contents can be correlated with the formation of alkali-resistant bounds occurring in cross-linked polymers in cell walls not extractable with the method commonly used to analyze phenolic acids (Iiyama et al., 1994).

Figure 3 also indicates a decrease in xantophylls and carotenes from the dough stage until maturity, in accordance with the pattern reported by Rodríguez-Suárez et al. (2014) and Sreenivasulu et al. (2010) for carotenoids in durum wheat and barley. Reduced levels in carotenes and xanthophylls during ripening may be due to their oxidation sensitivity, as a result of the high degree of unsaturation present in their structure. According to Mellado-Ortega and Hornero-Mendez (2016) and Sandmann et al. (2006), carotenes are likely to be more prone to oxidation than xanthophylls. This oxidation is caused by reactive oxygen species and especially singlet oxygen or free radicals generated by enzymatic systems such as lipoxygenases. Lipoxygenases catalyze the hydroperoxidation of polyunsaturated fatty acids, preferentially non-esterified polyunsaturated fatty acids, to form conjugated diene hydroperoxides (Loiseau et al., 2001). These hydroperoxides react with carotenoids, breaking down the carbon backbone into smaller compounds, including volatile molecules and apocarotenoids (e.g., epoxyaldehydes, ketones; Mellado-Ortega and Hornero-Mendez, 2016). Lipoxygenase is widely distributed in cereals and located in the germ and bran of the grain (Loiseau et al., 2001). A second explanation to the declining levels of carotenoids during grain ripening, could be linked to their esterification with fatty acids that produce mono and/or diesters and is catalyzed by xanthophyll acyltransferase enzymes. However, this second hypothesis is unlikely to occur based on the fact that xanthophyll esters seem to be absent or at very low levels in cereals and particularly in durum wheat (Mellado-Ortega and Hornero-Méndez, 2015).

Unlike kinetics of free and bound phenolic acids and carotenoids, the kinetic of tocopherols reported on Figure 3F indicates a gradual accumulation during the course of maize grain development. A similar pattern of total tocopherols was established by Gutierrez-Gonzalez et al. (2013) in oat seeds. According to the results of Falk et al. (2004) in developing barley kernels, tocopherols reach a maximum level at milk stage and remain stable until final harvest time. In rice, the total tocopherols in immature grains is about 2-fold higher than in mature ones (Lin and Lai, 2011).

Altogether, data describing the time course of *F. graminearum* infection and reporting the evolution of phytochemical levels in grains, provide evidence that the main antioxidant metabolites *F. graminearum* is likely to encounter when the production of mycotoxin starts *in planta* are free phenolic acids such as chlorogenic acid and bound ferulic and diferulic phenolic acids. Although present in lower concentrations, xantophylls, carotenes and benzoxazinoids, which show a high fungal toxicity, could also interfere with the fungus. Additional information on the impact antioxidant phytochemicals could exert on accumulation of mycotoxins in grains is provided by the results of recent studies that attempted to link plant resistance to *Fusarium* and antioxidant content of cereal grains (Siranidou et al., 2002; Bollina et al., 2011; Picot et al., 2013; Atanasova-Penichon et al., 2014).

## Relation between resistance to *Fusarium* spp. and antioxidant content in cereals

Host resistance is one of the primary traits that can be used as a control measure, and its manipulation is recognized as one of the best economic and ecological strategies to reduce damage caused by *Fusarium* (Bai and Shaner, 2004). Several authors argued that the use of cereal genotypes resistant to *Fusarium* infection (Champeil et al., 2004) and mycotoxin accumulation (Boutigny et al., 2008) is one of the most promising ways to reduce or prevent contamination. Combined with genetic approaches and the detection of QTL linked with FHB, GER, or FER resistance, biochemical ones aiming at deciphering the chemical mechanisms plants use to fight against *F. graminearum* and reduce toxin production hold great potential for assisting breeding programs (Gauthier et al., 2015). Most of the biochemical approaches that have addressed cereal resistance to *Fusarium* spp. and have been published to date, are based on a comparative analysis of the metabolite composition of resistant and susceptible cultivars, challenged or not with *Fusarium*. Targeted analytical tools and, more recently, metabolomics strategies were implemented. However, while these approaches can provide interesting insights that need to be further validated through genetic studies, they cannot allow conclusive evidence on the involvement of metabolite(s) or group(s) of metabolites in resistance. Indeed, the experimental designs frequently considered a set of limited and genetically unrelated genotypes and very rarely near isogenic lines, as done in the study of Gunnaiah et al. (2012). Moreover, data delivered through metabolomics approaches require to be considered with caution since differences in metabolic profiles of the studied genotypes may actually be confounding with the effects resulting of environment, cultivation practices and developmental stage. Lastly, it should be borne in mind that chemical identification remains a significant bottleneck in plant metabolomics studies and that most of the proposed identification are putative ones.

**Figure 4:**
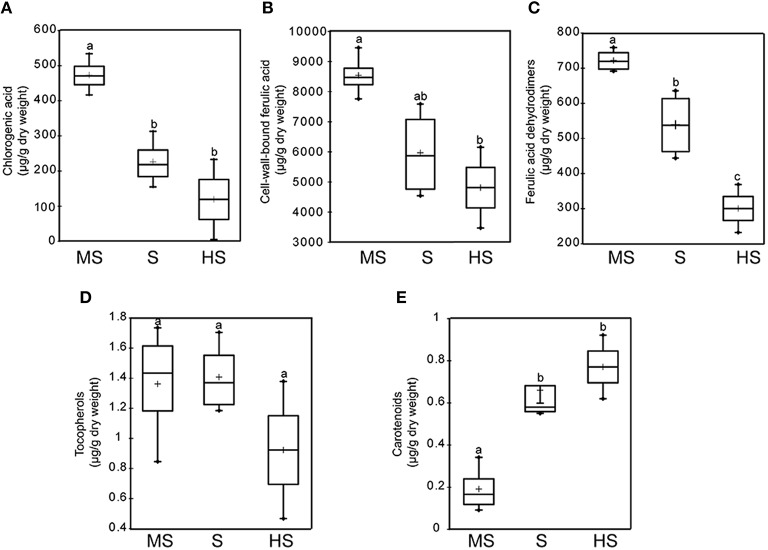
**Levels of some antioxidant metabolites in immature maize grains from three groups of varieties differing in their susceptibility to GER: highly susceptible (HS, *n* = 3), susceptible (S, *n* = 4), and moderately susceptible (MS, *n* = 3). (A)** chlorogenic acid; **(B)** cell-wall-bound ferulic acid; **(C)** ferulic acid dehydrodimers; **(D)** tocopherols and **(E)** carotenoids. Groups with different letters are significantly different. (Duncan, α = 0.05; Atanasova-Penichon et al., 2012; Atanasova-Penichon and Richard-Forget, 2014).

A number of studies focusing on phenolic acids supports the assumption that, in cereals, cell-wall-bound ferulic acid together with its dehydrodimers and free chlorogenic acid could be key components of the chemical defense against toxigenic *Fusarium* species (Siranidou et al., 2002; Atanasova-Penichon et al., 2012; Sampietro et al., 2013). Bily et al. (2003) highlighted that ferulic acid and its dehydrodimers in maize act as resistance factors to *F. graminearum* through type I resistance (resistance to initial penetration) and type II resistance (resistance to propagation due to a lower degradability of the cell wall). This hypothesis was further corroborated by Picot et al. (2013), Atanasova-Penichon and Richard-Forget (2014) and Atanasova-Penichon et al. (2014) who revealed the highest concentrations of ferulic acid, ferulic acid dehydrodimers and chlorogenic acid in immature grains of the more resistant varieties of a panel of maize genotypes with different susceptibility to FER or GER. Correlations between levels of GER resistance and phenolic acid contents in maize grains are reported on Figures 4A–C. When addressing maize resistance to GER, mechanisms resistance of silk that can slow down the process of infection need also to be addressed. Cao et al. (2011) have investigated the role of hydroxycinnamic acids in silk resistance and observed that, unlike data gathered in Figure 4, high concentrations in hydroxycinnamic acids were not related with a delayed progression of *F. graminearum* through silks. In wheat, positive relations between both free and cell wall bound phenolic acid levels and wheat resistance to FHB were reported by Siranidou et al. (2002). By the same reasoning, Choo et al. (2015) hypothesized that the high level of black barley resistance to FHB is linked to its richness in phenolic compounds. In addition to phenolic acids but with less conclusive evidence, many other phenylpropanoid compounds have been suggested to contribute to the chemical defense to FHB, GER, or FER. This potential contribution was mainly highlighted through comparative metabolomic profiling of grains issued from resistant and susceptible genotypes, challenged or not with toxigenic *Fusarium* strains (Hamzehzarghani et al., 2005, 2008; Browne and Brindle, 2007; Paranidharan et al., 2008; Bollina et al., 2010, 2011; Kumaraswamy et al., 2011a,b; Gunnaiah et al., 2012; Cajka et al., 2014; Chamarthi et al., 2014; Gunnaiah and Kushalappa, 2014). A large set of constitutive as well as inducible defense metabolites potentially related to *Fusarium* resistance was highlighted in the afore-mentioned studies. Among these metabolites, phenylpropanoids (approximately 180 compounds), including flavonoids and non-flavonoids, represent more than 50% of the total reported metabolites (Gauthier et al., 2015). Among these 180 phenylpropanoid candidates, more than 56% are putatively assigned as flavonoids that encompasse anthocyanins, flavones, flavonols, flavanones, flavanols, isoflavones, isoflavanones, isoflavonols, and chalcones. The remaining 44% is mainly composed by phenolic acids and derivatives, including benzoic and cinnamic ones.

Indeed, the role of phenylpropanoids in disease resistance has been the subject of intensive research (Treutter, 2006). In response to pathogen infection, they are released from the cell wall or massively synthesized by the plant accumulating rapidly at the site of infection (Nicholson and Hammerschidt, 1992). The main role ascribed to these compounds in plant defense mechanisms results from their antioxidant properties (Dykes and Rooney, 2007; Agati et al., 2012), that allow them to quench reactive oxygen species (ROS), generated by both the pathogen and the plant during infection. In addition, phenolic compounds operate in defense response through direct interference with the fungus, or through the reinforcement of plant structural components to act as a mechanical barrier against the pathogen (Siranidou et al., 2002; Treutter, 2006). Phenolic compounds such as flavonoids can also protect plant cell wall integrity upon fungal infection by inhibiting the activity of several plant cell wall degrading enzymes secreted by fungal pathogens to penetrate plant tissues (Treutter, 2005).

Concerning carotenoids and tocopherols, they are rarely regarded in comparative metabolomic studies due to their lipophilicity and the requirement of a specific extraction protocol and analytical equipment. While compilation of the metabolomic studies reported above results in a list of about 30 terpenoids (Gauthier et al., 2015), no carotenoid or tocopherol were among these terpenoid candidates. Targeted approaches aiming at relating lipophilic antioxidant composition of grains and resistance to *Fusarium* have also been implemented and showed positive or negative correlations, depending on the addressed group of compounds, carotenoids or tocopherols. Thus, based on the use of a set of maize genotypes with moderate to high susceptibility to GER, the experimentations we performed in our laboratory indicated higher levels of carotenoids (lutein + zeaxanthin + β carotene) in immature grains of the more susceptible genotypes (Figure 4E). A similar trend but not statistically significant was also observed between the level of FER resistance and carotenoid contents in maize grains (Picot et al., 2013). Accordingly, positive correlations between the levels of lutein and DON accumulation in durum wheat cultivars were reported by Delgado et al. (2014). As regard to tocopherols, while an absence of correlation between resistance levels of maize and their tocopherol contents in immature grain was observed by Picot et al. (2013), the data of Iqbal et al. (2014) showed the existence of a negative correlation between the concentrations of tocopherols and aflatoxins in rice cultivars. Interestingly, the report of Boba et al. (2011), based on the use of transgenic flax overproducing carotenoids, indicates that a general level of lipophilic antioxidants rather than the content of any particular compound is the most important factor in resistance to *F. culmorum* infection. The main role ascribed to carotenoids and tocols in plant/*Fusarium* interactions directly results from their ability to quench the free radicals produced by plant cells (the so-called “oxidative-burst”) as a first response to the fungal pathogen attack (Boba et al., 2011; Gutierrez-Gonzalez et al., 2013). Moreover, carotenoids are directly linked to abscisic acid, which level in wheat and barley was shown to increase after *F. graminearum* or *F. culmorum* inoculation (Gunnaiah et al., 2012; Kumaraswamy et al., 2012; Petti et al., 2012). In fact, abscisic acid is an apocarotenoid synthetized from the cleavage of carotenoids (Tan et al., 1997). 9-cis-epoxycarotenoid dioxygenases cleave 11,12 double bonds of the cis isomers of violaxanthin and neoxanthin to form the C15 product xanthin, the first committed and key regulatory step in the abscisic acid biosynthesis pathway (Sreenivasulu et al., 2010). Abscisic acid is well known for its roles in orchestrating stress response as well as grain maturation in plants. In addition, its role in resistance of wheat to FHB has been linked to a regulatory effect on callose deposition in the transition zone between the spikelet's rachilla and rachis. This was shown to contribute to the type II resistance (Kang and Buchenauer, 2000; Flors et al., 2005). Besides, the involvement of abscisic acid in FHB resistance has also been ascribed to its negative interaction with the signaling ethylene pathway (Flors et al., 2005) since, according to Chen et al. (2009), *F. graminearum* can exploit ethylene signaling to enhance colonization in wheat tissues. Lastly, the possibility that abscisic acid could limit *F. graminearum* penetration through its control of stomatal aperture cannot be omitted (Mauch-Mani and Mauch, 2005).

As regards to benzoxazinoids, while several reports have indicated their ability to inhibit fungal activities linked with FER and GER (Miller et al., 1996; Glenn et al., 2001; Etzerodt et al., 2015), very few studies have investigated their concentration in cereals in relation to *Fusarium* sensitivity. One study (Søltoft et al., 2008) has revealed positive correlations between the susceptibility of wheat to FHB and the concentrations of some benzoxazinoid derivatives, suggesting that the capacity of wheat to produce these secondary metabolites could contribute to resistance mechanisms. However, as emphasized above, the results of Søltoft et al. (2008) based on the use of a set of unrelated germplasms are not sufficient to draw conclusive evidences on the involvement of benzoxazinoids in FHB resistance.

## Conclusion

Cereal diseases caused by pathogenic and toxigenic *Fusarium* species are responsible for major economic damage worldwide. Hence, the developments of sustainable strategies to avoid *Fusarium* and mycotoxin contamination have been the issue of intense research over past years and decades and a broad consensus has emerged to acknowledge that the use of FHB, GER or FER resistant genotypes is one of the primary pillars of any disease management programs. However, to date, knowledge of the complex mechanisms governing cereal resistance remains insufficient, and selection for resistant genotypes is still challenging.

Considering the available data on the interactions between antioxidant metabolites in grains and *Fusarium* species, we can assume that some of these compounds could significantly contribute to the protection of grains against toxigenic *Fusaria* and mycotoxin accumulation. Five main classes of antioxidant metabolites, phenolic acids, flavonoids, carotenoids, tocopherols and benzoxazinoids, have been evidenced for the pivotal role they could play in FHB, GER, or FER resistance. A first shared argument in favor of the involvement of phenolics, carotenoids and tocopherols is linked to their ability to quench reactive oxygen species, thus protecting biological cells. In addition, tocopherols and carotenoids have the capacity to scavenge lipid peroxyl free radical and therefore to stop the chain propagation of the lipid peroxidation cycle (Das and Roychoudhury, 2014). A second shared argument rests on the fungal toxicity exhibited by cereal antioxidant metabolites. Indeed, as demonstrated by the present review, there are numerous studies illustrating the efficiency of phenolic compounds, carotenoids, tocopherols and even benzoxazinoids to restrain the growth of toxigenic *Fusaria* and their production of toxins. Lastly, phenolic compounds are known to participate to the reinforcement of plant structures and contribute therefore to the establishment of a physical barrier against fungal infection.

However, while involvement of antioxidant metabolites in resistance mechanism to *Fusarium* spp. has been highly suggested, this involvement is far from being elucidated. One major challenge for the coming years will be to obtain conclusive proofs. Even though the genetic architecture underlying the synthesis and regulation of secondary metabolites in cereals is extremely complex, pieces of evidence can certainly come from extensive genetic and functional genomic studies.

## Author contributions

VA and FR have made substantial, direct and intellectual contribution to the work. CB has made intellectual contribution. All authors approved this work for publication.

### Conflict of interest statement

The authors declare that the research was conducted in the absence of any commercial or financial relationships that could be construed as a potential conflict of interest.
